# The evolving contribution of MRI measures towards the prediction of secondary progressive multiple sclerosis

**DOI:** 10.1136/jnnp-2024-333917

**Published:** 2024-07-22

**Authors:** Piriyankan Ananthavarathan, Nitin Sahi, Karen Chung, Lukas Haider, Ferran Prados, S Anand Trip, Olga Ciccarelli, Frederik Barkhof, Carmen Tur, Declan T Chard

**Affiliations:** 1 NMR Research Unit, Queen Square MS Centre, Department of Neuroinflammation, UCL Queen Square Institute of Neurology, Faculty of Brain Sciences, University College London, UK; 2 Department of Biomedical Imaging and Image Guided Therapy, Medical University of Vienna, Wien, Wien, Austria; 3 Centre for Medical Image Computing (CMIC), Department of Medical Physics and Biomedical Engineering, University College London, London, UK; 4 e-Health Centre, Universitat Oberta de Catalunya, Barcelona, Spain; 5 Department of Radiology & Nuclear Medicine, Amsterdam UMC, Vrije Universiteit, the Netherlands; 6 Multiple Sclerosis Centre of Catalonia (Cemcat). Vall d’Hebron Barcelona Hospital Campus. Barcelona, Spain; 7 National Institute for Health Research (NIHR) University College London Hospitals Biomedical Research Centre, UK

**Keywords:** multiple sclerosis, neurodegeneration, inflammation, pathogenesis, clinical outcomes, predictive models

## Abstract

**Background:**

In multiple sclerosis (MS), both lesion accrual and brain atrophy predict clinical outcomes. However, it is unclear whether these prognostic features are equally relevant throughout the course of MS. Among 103 participants recruited following a clinically isolated syndrome (CIS) and followed up over 30 years, we explored: (1) whether white matter lesions were prognostically more relevant earlier and brain atrophy later in the disease course towards development of secondary progressive disease; (2) if so, when the balance in prognostic contribution shifts; and (3), whether optimised prognostic models predicting secondary progressive disease should include different features dependent on disease duration.

**Methods:**

Binary logistic regression models were built using age, gender, brain lesion counts and locations, and linear atrophy measures (third ventricular width [TVW] and medullary width [MEDW]) at each timepoint up to 20 years, using either single timepoint data alone or adjusted for baseline measures.

**Results:**

By 30 years, 27 participants remained CIS, while 60 had MS (26 SPMS, 16 MS-related death). Lesions counts were prognostically significant from baseline and at all later timepoints, while linear atrophy measure models reached significance from 5 years. When adjusted for baseline, in combined MRI models including lesion count and linear atrophy measures, only lesion counts were significant predictors. In combined models including relapse measures, expanded disability status scale (EDSS) scores and MRI measures, only infratentorial lesions were significant predictors throughout.

**Conclusions:**

While SPMS progression is associated with brain atrophy, in predictive models only infratentorial lesions were consistently prognostically significant.

## Introduction

Multiple sclerosis (MS) is clinically highly heterogenous. While some people accrue substantial disability or have a significantly shortened lifespan^1^, others develop few detectable long-term neurological deficits^2^. After the first clinical event (a clinically isolated syndrome, CIS), most (~85%) run a relapsing-remitting (RRMS) disease course^3^ with many subsequently transitioning to secondary progressive (SP)MS (~50% within 15-20 years)^4,5^. It is during SPMS that individuals acquire most disability.^3,6,7^ There is growing evidence that earlier treatment reduces the risk of, or at least significantly delays, SPMS onset^8,9^ and hence there is a trend towards treating MS earlier with higher efficacy agents. However, given that a significant proportion may not develop clinically progressive disease or substantial disability^10,11^, and the potential for serious harm from disease modifying treatments (DMT), in addition to those with clinically active disease, early use of high efficacy agents would ideally be weighted towards those at the clearest risk of developing SPMS.

Clinical factors associated with a more aggressive MS phenotype include older age at initial presentation, early frequent relapses, and shorter intervals between first and second relapses^2,12,13^. Presenting with an optic neuritis or sensory-predominant CIS has also been linked to a less disabling clinical course, although debated^2,12,14^. Radiological features associated with an aggressive MS phenotype include higher numbers^15^ and volumes of white matter^16,17^ (WM) and grey matter^18,19^ (GM) lesions, the presence of posterior fossa and spinal cord lesions^10^, and faster rates of brain atrophy.^20^ Transitioning from RRMS to SPMS is associated with declining WM lesion formation and increasing brain atrophy^21^, although accelerated brain atrophy still occurs early in MS, particularly among people who eventually develop SPMS^21^. Consistent with this, disability early in RRMS is mainly thought to be due to relapse activity and WM lesion accrual^10,17^, although there is growing recognition that substantial progression independent of relapses may also occur in RRMS^22^. In established SPMS, disability relates more closely to brain atrophy^20,23^. It can therefore be hypothesised that, when predicting SPMS development, WM lesion accrual is more relevant earlier while brain atrophy increases in relevance closer to SPMS onset. However, we have previously shown that brain atrophy independently contributes to prognostic models within the first 5 years after symptom onset^20^, so this hypothesis may be incorrect. Systematically investigating prognostic markers for SPMS is difficult as it typically develops 15 or more years after first symptoms onset^24,25^, and thus long-term follow-up is required to test this.

We previously reported on a 30-year longitudinal follow-up study of 107 participants presenting with a CIS (by 30 years 28% (n=30) remained CIS, 32.7% (n=35) had RRMS, while 39.3% (n=42) had either SPMS or died due to MS), where we explored the prognostic significance of lesion numbers, location and linear brain atrophy measures in the first 5 years after symptom onset^10,20^. People who transitioned from RRMS to SPMS did so ~17 years after symptom onset and both lesion accrual (in particular, infratentorial lesions) and brain atrophy (measured using medullary width [MEDW], but not third ventricular width [TVW]) predicted SPMS development by 30 years^20^.

In the present study, we sought to answer three questions: (1) Are WM lesions more prognostically relevant early, and brain atrophy measures later, in the disease course? (2) If so, how long after disease onset does the balance between them shift? and (3), should optimised prognostic models include different features dependent on disease duration? The main clinical outcome considered was the development of SPMS but to test consistency we also explored other outcome measures (MS-related mortality, and EDSS ≥3.5 by 30 years).

We undertook this with a view to clinical practice, where volumetric brain atrophy measures are not, for practical reasons, routinely assessed. Given that in clinical practice serial scans are often unavailable or acquired using very different machines and protocols, we ran both cross-sectional analyses (using single timepoint data) and longitudinal analyses to determine the added value of serial scanning.

## Materials and Methods

### Study Participants

The clinical characteristics of this cohort have been previously described.^10^ 140 participants were prospectively recruited between 1984-1987 after first presenting with a CIS.^26^ Participants underwent radiological (MRI) assessment at 1 year (n=108), with clinical and radiological assessments at 5(n=92), 10(n=66), 14(n=55), 20(n=75) and 30(n=63) years.^10,20^ All participants provided informed consent to take part in the study.

Eight participants subsequently found to have a diagnosis other than CIS or MS and were excluded, and by 30 years clinical outcomes were known among 120 and were assessed using the 2010 revised McDonald clinical and MRI criteria.^27^ Thirteen participants who died from unrelated causes by 30 years were excluded due to uncertainty in neuroinflammatory outcomes following a CIS. One participant included in our analysis had a diagnosis of idiopathic Parkinson’s disease and remained CIS throughout; the remaining cohort had no other known neurodegenerative diseases. Among 107 remaining participants, four (three CIS, one RRMS) had missing or inadequate baseline, 1- or 5-year scans and were excluded from analysis. 103 participants were ultimately included in the present analysis: by 30 years, 27 (26.2%) remained CIS, 34 (33.0%) had RRMS, 26 (25.2%) had SPMS, while 16 (15.5%) had died due to MS (preceded by an SPMS course).

### Clinical Assessment

Expanded Disability Status Scores^28^ (EDSS) and clinical relapse frequency were determined at baseline, 5, 10, 14, 20 and 30-year visits. Baseline EDSS was calculated retrospectively from review of notes, while 30-year EDSS assessments were undertaken either in person (66 participants) or telephone (25 participants). Where participants were not assessed at a given timepoint, EDSS was inferred from available clinical data and EDSS at adjacent timepoints. 14 participants did not have baseline EDSS retrospectively calculated as these could not be confidently calculated from available records.

### Image Acquisition

Image acquisition and analysis protocols have been previously described in detail elsewhere^10,20^. Baseline, 1-year and 5-year timepoint MRI scans were obtained using a 0.5T Picker system (Marconi Medical Systems, Cleveland, OH). 10-, 14- and 20-year timepoint MRI scans were obtained using a 1.5T General Electric Signa system (GE Healthcare, Chicago, IL), while a 3T Philips Achieva system (Philips Healthcare, Best, The Netherlands) was used at the 30-year time point. At each time point, proton density (PD) and/or T2-weighted images were acquired. Baseline, 1-year and 5-year film images were digitised using Vidar Diagnostic Pro Advantage film digitizer (VIDAR Systems, Herndon, VA)^10,29^.

Lesions were marked and their location assessed (juxtacortical [JC], periventricular [PV], infratentorial [IT], and deep white matter [DWM])^10^. As baseline, 1-, 5- and 10-year images were not suitable for volumetric MRI analysis, linear atrophy measures were employed. Third ventricular width (TVW) was measured by drawing a midpoint line running parallel to the long axis of the ventricle on axially acquired PD/T2-weighted MRI scans^20,30^. Medullary width (MEDW) was measured as the dorsoventral medullary diameter on midsagittal imaging (scout images at baseline, 1 and 5 years, and T1-weighted images at subsequent time-points)^20,31^.

### Statistical Analysis

Statistical analyses were performed using IBM SPSS Statistics v28.0.0 (IBM Corporation, Armonk, NY).

To answer our questions on the prognostic relevance of WM lesions and brain atrophy measures over time, and whether there was a shift in the balance between them, we built cross-sectional binary logistic regression models using MRI measures from each timepoint. Using the Nagelkerke method, a pseudoR^2^ value (pR^2^) was calculated as a measure of model fit and the strength of contributory effect (association) of a particular measure towards a given outcome. Models predicting SPMS development were repeated with and without age and gender as additional covariates, with the difference in pR^2^ between the two models calculated to determine the sole contribution of each MRI measure over time. To determine the longitudinal effects of MRI measures, models were reran adding their respective baseline MRI measures. Models were not censored: all participants who reached a particular outcome (e.g. SPMS) by an earlier timepoint were included in all subsequent timepoint models (to avoid biasing later models by increasingly including only people at lower risk of SPMS over time).

As TVW increases with brain atrophy, while MEDW decreases, we present the inverse measure of MEDW (1/MEDW). Linear atrophy models adjusted for baseline are statistically equivalent to modelling the rates of atrophy from baseline. However, for completeness, further models were ran using calculated atrophy rates (see Supplementary Materials).

To determine if prognostic models should include different features dependent on disease duration, we built longitudinal binary logistic regression models using a forward and backward conditional approach including all MRI measures, to determine which covariates were retained for optimal models at each timepoint. Here, we present the full model’s pR^2^, odds ratio (OR) and the significance of each covariate. With a view to clinical applicability, we manually built additional combined models incorporating both MRI and clinical (age, gender, relapse frequency, EDSS) measures to determine which most optimally predicted ultimate clinical outcomes at each timepoint. The base model started with age and gender, to which we sequentially added MRI and clinical measures, and observed which gave the highest overall pR^2^.

We present the results of models predicting SPMS below (models predicting MS-related mortality or EDSS ≥3.5 outcomes are presented in Supplemental Materials).

A p-value ≤0.05 was considered statistically significant.

## Results

We present model pR^2^ below (full results in Supplementary Materials). For context, models predicting SPMS based on age (at first CIS) and gender alone had a pR^2^=0.029.

### Lesion accrual predicting SPMS

#### Whole brain lesion counts

**Figure 1:**
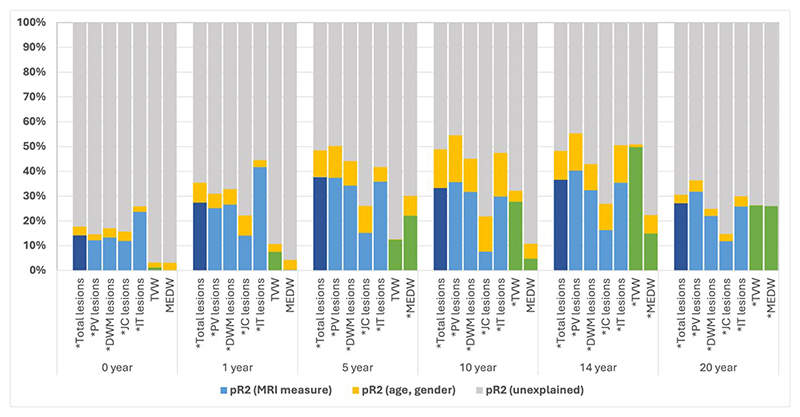
Cross-sectional models considering contribution of age, gender and MRI measures (total lesion count, lesion by subtype, linear atrophy measures) towards 30-year SPMS For all figures, * indicates a model that was significant at p≤0.05. At some timepoints age and gender explained small amounts of the outcome of interest and may therefore not be clearly seen in the Figures. Please see Tables 4 & 5 in supplementary materials for a detailed breakdown of the values.

**Figure 2:**
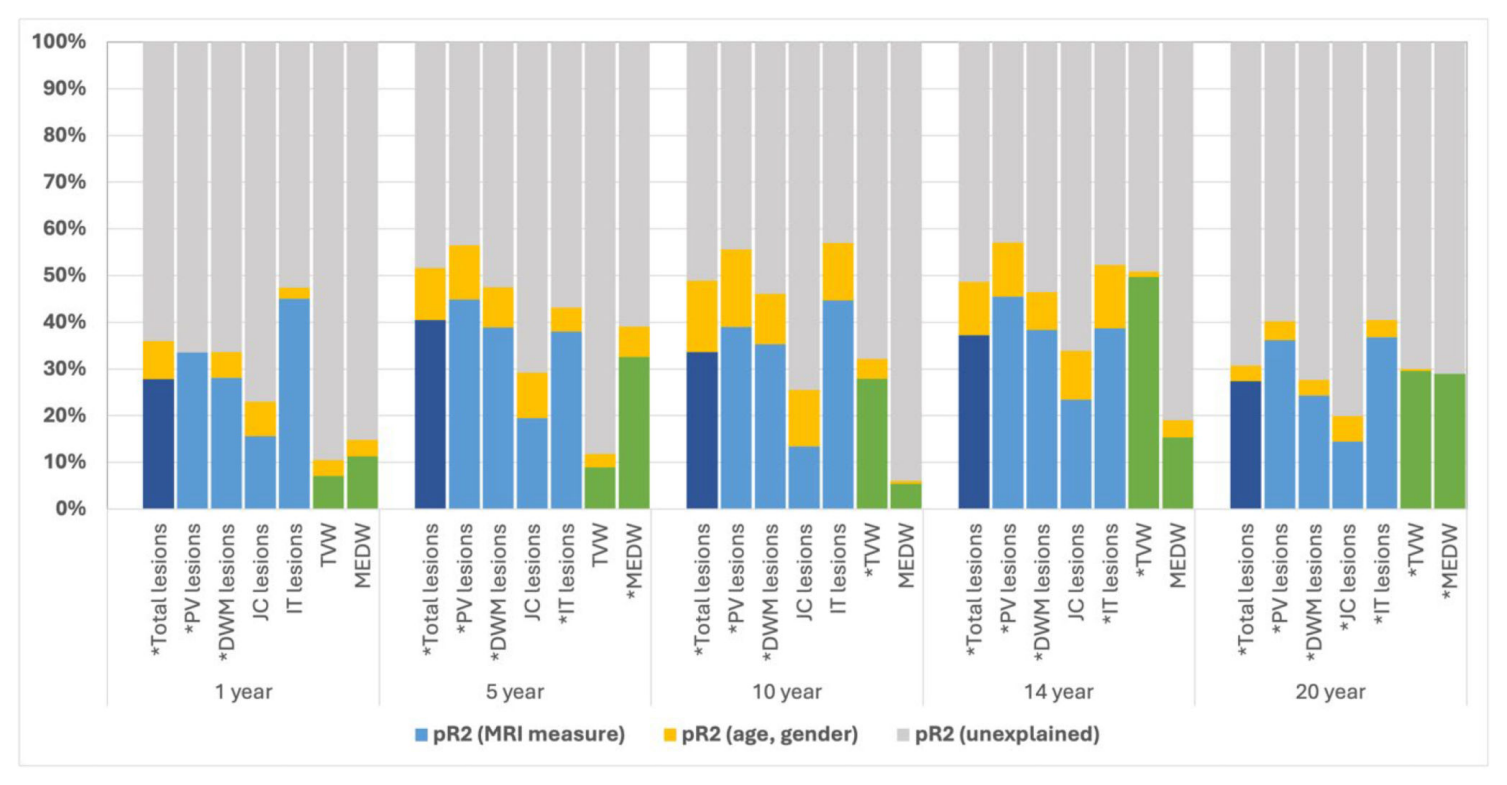
Longitudinal models considering contribution of age, gender and MRI measures (total lesion count, lesion by subtype, linear atrophy measures) towards 30-year SPMS

In cross-sectional models predicting SPMS, whole brain lesion counts were significant at all timepoints (peak contributory effect at 5 years, pR^2^=0.377). Contributory effects increased over the first 5 years, then plateaued up to the 14-year timepoint before declining at 20 years. A similar pattern was observed in longitudinal models which included baseline whole brain lesion counts (Figures 1 and 2, and Supplementary Table 4).

#### Lesion counts by location

Total IT lesion counts peaked in contributory effect at the 1-year timepoint (pR^2^=0.417) in cross-sectional models, after which they plateaued before declining at the 20-year timepoint. Total IT lesion count was a significant variable across all cross-sectional models. In longitudinal models (adjusted for baseline IT lesion counts), total IT lesion count was a significant variable at 5- and 14-year timepoints with similar contributory effects (Figures 1 and 2, and Supplementary Table 5).

Cross-sectional models considering either PV or DWM lesion counts both showed increasing contributory effects over the first 5 years, after which their effects plateaued before subsequently declining at 20 years (peak pR^2^=0.403 at 14 years for PV models, peak pR^2^=0.342 at 5 years for DWM lesion count models). Similar trajectories were noted for longitudinal models adjusted for baseline PV or DWM lesion counts (Figures 1 and 2). PV and DWM lesion counts remained significant variables across all timepoints in cross-sectional and longitudinal models.

JC lesion count models had the lowest predictive ability towards development of SPMS (peak pR^2^=0.152 in cross-sectional models), although significant across all timepoints. In longitudinal models adjusted for baseline, total JC lesion counts were only significant at 20 years (Figures 1 and 2).

### Linear atrophy predicting SPMS

#### Third ventricular width (TVW)

In cross-sectional models, TVW increased in contributory effect of over time, peaking at 14 years (pR^2^=0.498). A similar trend was observed in longitudinal models (Figures 1 and 2). TVW was a significant variable in cross-sectional and longitudinal models from the 10-year timepoint onwards.

#### Medullary width (MEDW)

While MEDW was a significant variable in cross-sectional models at 5, 14 and 20-year timepoints, its predictive power was greatest at the 5- and 20-year timepoints (pR^2^=0.221 and 0.260 respectively). A similar trend was observed in longitudinal models adjusted for baseline MEDW, where greatest contributory effect was observed at 5- and 20-year timepoints (and were the only timepoints where MEDW was a significant model variable) (Figures 1 and 2).

### Clinical factors predicting SPMS

Relapse frequency was significant in cross-sectional models across all timepoints, with peak associations observed between 5-10 years (pR^2^=0.213). Conversely, EDSS increasingly associated with SPMS outcomes over time in cross-sectional models (peak effects at 20 years, pR^2^=0.688) and was a significant variable from the 5-year timepoint onwards (see Supplementary Table 6 for full results).

### Optimal models predicting SPMS

#### MRI features alone (Tables 1 & 2)

In combined cross-sectional models (Table 1), at baseline, only IT lesion counts were retained (and significant) (model pR^2^=0.174). At the 5-year timepoint, combined models included age, 5-year PV lesion counts and 5-year MEDW (model pR^2^=0.595; age and 5-year PV lesion counts were significant variables). At the 10-year timepoint, combined models included 10-year IT lesion counts and 10-year MEDW (model pR^2^=0.504; both 10-year IT lesion counts and 10-year MEDW were significant variables). At the 14-year timepoint, combined models only included 14-year TVW and was a significant model variable (model pR^2^=0.535). By the 20-year timepoint, the model included 20-year DWM lesion counts and 20-year TVW (model pR^2^=0.439; both 20-year DWM lesion counts and 20-year TVW were significant variables).

In combined longitudinal models (including baseline MRI measures, age and gender) (Table 2), at the 5-year timepoint, the model retained 5-year PV lesion counts and 5-year MEDW (model pR^2^=0.525; 5-year PV lesion count was a significant variable). At the 10-year timepoint, only baseline IT lesion count was retained in combined longitudinal models and was a significant variable (model pR^2^=0.391). At the 14-year timepoint, combined longitudinal models only retained 14-year PV lesion counts and was a significant variable (model pR^2^=0.475). At 20-years, models retained both baseline IT lesion counts and 20-year TVW (model pR^2^=0.555; baseline IT lesion counts was a significant variable).

#### MRI and clinical features (Table 3)

At the 5-year timepoint, combined models retained age, gender, relapse activity between 0-5 years and 1-year IT lesion counts (model pR^2^=0.484; age, relapse activity between 0-5 years and 1-year IT lesion counts were significant model variables). At the 10-year timepoint, age, gender, clinical relapse activity between 5-10 years, 1-year IT lesion counts and 10-year EDSS were retained in the model (model pR^2^=0.663; 10-year EDSS, relapse activity between 5-10 years, and 1-year IT lesion counts were significant model variables); while at the 14-year timepoint, age, gender, clinical relapse activity between 5-10 years and 14-year IT lesion counts were retained (model pR^2^=0.642; age, 14-year IT lesion counts and relapse activity between 5-10 years were significant model variables). At the 20-year timepoint, combined models retained age, gender, 1-year IT lesion counts and 20-year EDSS (model pR^2^=0.729; 20-year EDSS and 1-year IT lesion counts were significant model variables).

## Discussion

We found that WM lesions were prognostically relevant for the development of SPMS immediately after a CIS and, contrary to our initial hypothesis, remained clinically relevant throughout follow-up. In combined MRI models, linear atrophy measures increasingly contributed towards predictive power at later follow-up points, but when adjusted for baseline MRI measures, only WM lesion counts remained significant. Similarly, when clinical features were introduced into models, among MRI measures, only IT counts remained significant predictors.

Lesion counts had greatest prognostic relevance from 5 to 14 years, whilst linear atrophy measures had a more complex relationship with outcomes. TVW was most significantly predictive at 14 years (reaching significance from 10 years), while MEDW was most significantly predictive at 5 and 20 years. While overall this is consistent with lesion accrual being of slightly diminishing clinical relevance over time and atrophy becoming increasingly important^10,20^, when models were built with both lesion counts and linear atrophy measures alongside clinical features, only lesion counts independently contributed to the prediction of SPMS. While brain atrophy has been shown to correlate better than lesion accrual with disability in established progressive MS^21,23^, our results suggest that linear atrophy measures have lesser value in predicting SPMS onset. In line with previous work^10,32^, we found lesion location influenced prognostic relevance, with IT and PV lesions showing similar pR^2^ and higher than that observed in DWM and JC lesions respectively. Only IT lesion counts contributed significantly to models that also included clinical measures. Even 20 years after initial CIS, IT lesions continued to have significant prognostic relevance for SPMS by 30 years. While lesion location is clearly relevant to symptoms during a relapse, it remains unclear why lesion location also influences overall risk of progressive MS. The prognostic significance of TVW increased over time from 5 years, reaching peak contributory effect at 14 years. Medullary thinning had a more complex association with SPMS development, peaking at both 5 and 20 years. It is worth recalling that both TVW and MEDW are regional measures of atrophy: TVW correlates most with brain parenchymal fractions (r=-0.93 at 30-year scanning), whereas MEDW correlates most with cord volumes (r=0.61 at 30-year scanning)^20^, and atrophy due to MS preferentially affects different regions of the brain^33^ and spinal cord^34^ at different stages of disease.

When adjusted for baseline values, MEDW was not a consistent predictor of SPMS. This may be explained by the changes in scanners and scanning protocols which occurred over time (leading to step changes in all measures), measurement noise (initially 2D non-isotropic scans were used, obtained at 0.5T with a 5 mm slice thickness), and differing numbers of participants at each timepoint. However, while these factors will obscure associations, they will not lead to spurious ones being found.

As volumetric atrophy measures could not be applied to early MRI data, we used linear approaches. Compared to volumetric measures they are much easier to undertake, but for the reasons noted earlier we think that associations with clinical outcomes may have been attenuated. In particular, the low resolution of early scans in this study will have been associated with higher partial volume effects when compared with current scans (now very often isotropic and close to 1x1x1mm). Volumetric atrophy measurement approaches can be applied to modern scans, but have a significant computational overhead, and so linear methods may still be more feasible in clinical practice. Given the results of our study, it would be interesting to compare the sensitivity to change of volumetric and linear measures using current routinely obtained clinical scans.

Another consequence of technical advances since the start of this study was the limited acquisition of other MRI measures that may be of interest in predicting progressive MS, specifically imaging the spinal cord for lesions and measuring atrophy^34,35^, or dedicated brain imaging to detect grey matter lesions (which, in the present cohort, was the MRI feature that most distinguished SPMS from RRMS at 30 years^18^). However, none of these are routinely acquired in current clinical practice and does not undermine the relevance of the current findings, although would be of interest in future studies.

It is important to also note that MRI data availability differed between timepoints: for example, at baseline MRI data was available for 103 participants, while at 14 years 52 were scanned. Models based on smaller sample sizes will have less power to detect associations, and so factors not found to be statistically significant in this study may still be clinically relevant (and might have probably been statistically significant if larger sample sizes had been analysed), albeit less so than those where a predictive effect was detected.

Given that we specifically sought to investigate the prognostic relevance of MRI measures towards 30-year outcomes (rather than their direct correlation with disability accrual over time), we used binary logistic regression models to identify which factors significantly contributed towards prognostic power for a given timepoint. However, it is worth noting that there are both biological (e.g., WM lesions disrupting tracts, leading to neurodegeneration and disability) and temporal (WM lesion load, brain atrophy and disability all naturally increase over time) reasons for collinearity between measures, and hence, models may be dominated by a particular measure with the strongest association towards an outcome at a given time. This does not necessarily mean that other measures are irrelevant to clinical progression *per se*, but rather, they did not contribute to a given prognostic model.

Despite brain atrophy measures increasing in prognostic relevance over time in isolation, they also did not contribute significantly to models including WM lesion and clinical features. While brain atrophy is associated with SPMS, it is also predicted to a degree by preceding WM lesion accrual, and in part it may be argued that atrophy simply reflects a later stage in a pathological cascade from WM lesion formation to tract-mediated damage and eventual brain atrophy (for example^36^). Furthermore, there is growing evidence that with progressive MS, while WM lesion accrual slows, as many as ~30% of lesions transition towards chronic activity^37^, and chronic demyelination is also associated with ongoing axonal loss^38^. Based on this, the ultimate effect of early WM lesion accrual on neurodegeneration may take years to manifest (and in previous work with this cohort, associations strengthened over more than a decade^39^).

As expected, relapse activity (as a clinical predictor of 30-year outcomes) followed similar trends to lesion counts over time, increasing in predictive ability up to 5-10 years from first symptom onset, after which its effects progressively diminished. As would be expected based on brain atrophy measures, EDSS also significantly predicted SPMS, increasing in predictive ability from 5 years, although it is important to note that higher EDSS scores per se will increasingly distinguish RR from SPMS, and in combination with other features, has been used as part of an objective definition of SPMS^40^.

Our study benefited from 30 years of clinical and radiological data, with clear phenotypic separation by the end of the follow-up period. Licensed DMTs were unavailable when participants were recruited and only introduced a decade or more later, hence most were untreated. Only eleven people (four RRMS, seven SPMS at 30 years) received a DMT at any point, the earliest starting 10 years after MS diagnosis. While this offered a unique insight into the natural history of MS disease progression, it cannot be assumed that MRI prognostic features identified in this cohort are as relevant among people taking current high-efficacy treatments.

In conclusion, while we presupposed a shift between the prognostic relevance of WM lesion accrual and brain atrophy, our results suggest that IT lesions consistently remained prognostically significant towards the development of SPMS in combined clinical and MRI models.

## Supplementary Material

Supplementary Material

## Figures and Tables

**Table 1:** Lean cross-sectional MRI models (lesions by location, age, gender) predicting 30-year SPMS

Timepoint	n	Model pR^2^	Model covariates	Covariate OR (95% CI)	Covariate p=
**0 year**	**58**	**0.174**	**0-year IT lesion count**	**2.31 (1.13-4.71)**	0.020*
**5 year**	**61**	**0.595**	**Age**	**1.14 (1.01-1.28)**	0.030*
			**5-year PV lesion count**	**1.40 (1.15-1.70)**	<0.001*
			5-year MEDW	0.36 (0.13-1.02)	0.055
**10 year**	**46**	**0.401**	**10-year IT lesion count**	**1.80 (1.16-2.80)**	0.009*
			**10-year MEDW**	**0.36 (0.12-1.05)**	0.060*
**14 year**	**38**	**0.535**	**14-year TVW**	**2.48 (1.38-4.44)**	0.002*
**20 year**	**52**	**0.439**	**20-year DWM lesion count**	**1.02 (1.00-1.05)**	0.036*
			**20-year TVW**	**1.48 (1.05-2.10)**	0.026*

**Table 2:** Lean longitudinal MRI models (lesions by location, age, gender) adjusted for baseline MRI measures predicting 30-year SPMS

Timepoint	n	Model pR^2^	Model covariates	Covariate OR (95% CI)	Covariate p=
**5 year**	**43**	**0.525**	**5-year PV lesion count**	**1.27 (1.08-1.51)**	0.005*
			5-year MEDW	0.26 (0.07-1.02)	0.053
**10 year**	**27**	**0.391**	**0-year IT lesion count**	**9.25 (1.03-82.9)**	0.047*
**14 year**	**22**	**0.475**	**14-year PV lesion count**	**1.20 (1.03-1.39)**	0.017*
**20 year**	**31**	**0.555**	**0-year IT lesion count**	**9.61 (1.15-80.5)**	0.037*
			20-year TVW	1.60 (1.00-2.59)	0.052

**Table 3:** Lean combined models (clinical and MRI) predicting 30-year SPMS

Timepoint	n	Model pR^2^	Model covariates	Covariate OR (95% CI)	Covariate p=
5 year	78	0.484	**Age**	**1.10 (1.00-1.20)**	0.042*
			Gender	0.70 (0.19-2.52)	0.581
			**Number of relapses (0-5 years)**	**1.59 (1.08-2.33)**	0.018*
			**1-year IT lesion counts**	**4.25 (1.62-11.12)**	0.003*
10 year	70	0.633	Age	1.12 (1.00-1.26)	0.053
			Gender	0.19 (0.03-1.23)	0.081
			**10-year EDSS**	**2.09 (1.21-3.62)**	0.008*
			**Number of relapses (5-10 years)**	**2.44 (1.20-4.99)**	0.014*
			**1-year IT lesion counts**	**5.38 (1.22-23.67)**	0.026*
14 year	43	0.642	**Age**	**1.23 (1.03-1.47)**	0.023*
			Gender	0.10 (0.01-1.01)	0.051
			**14-year IT lesion counts**	**1.92 (1.16-3.17)**	0.011*
			**Number of relapses (5-10 years)**	**2.75 (1.16-6.48)**	0.021*
20 year	82	0.729	Age	1.04 (0.93-1.15)	0.526
			Gender	0.64 (0.13-3.06)	0.572
			**20-year EDSS**	**2.40 (1.57-3.69)**	<0.001*
			**1-year IT lesion counts**	**6.89 (1.83-25.91)**	0.004*

## Data Availability

Anonymised data which is not published in the article can be shared on reasonable request from a qualified investigator.

## Key messages

### What is already known on this topic

Both MRI measures of white matter lesion accrual and atrophy predict clinical outcomes in people with multiple sclerosis. However, it is unclear whether both are equally relevant in predicting long-term outcomes (20 years or more) following a first relapse and, in particular, the development of secondary progressive disease. We investigated this, anticipating that white matter lesions may be more relevant earlier, and atrophy measures later, into the disease course.

### What this study adds

We found that, while brain atrophy (measured using linear distance between features visible on conventional MRI scans and hence feasible in routine clinical practice) was prognostically relevant to the prediction of secondary progressive disease later in the clinical course of multiple sclerosis, lesion counts were of early and enduring relevance over time. When considered alongside clinical features in predictive models, the only MRI feature that clearly contributed was the presence of infratentorial lesions.

### How this study might affect research, practice, or policy

Our findings reinforce previous observations that infratentorial lesions predict worse clinical outcomes and onset of secondary progressive disease, whilst further demonstrating their prognostic relevance even up to 20 years after first symptom onset. They also suggest that linear brain atrophy measures have little to add as prognostic markers in clinical practice.
