# Supplementary material for: The evolving contribution of MRI measures towards the prediction of secondary progressive multiple sclerosis
Source: J Neurol Neurosurg Psychiatry. Author manuscript; Available in PMC 2025 Sep 1. (PMC7616528; doi:10.1136/jnnp-2024-333917)
Supplement: Supplementary Material [file EMS197922-supplement-Supplementary_Material.pdf]

## Supplementary Material

## Table 4 – Total lesion count and linear atrophy models predicting 30-year SPMS outcomes

**Table 4a – Cross-sectional models predicting 30-year SPMS outcomes**
*Adjusted for age and gender*

| Timepoint | Total Lesion Counts | | | | | | TVW | | | | | | MEDW | | | | | |
|---|---|---|---|---|---|---|---|---|---|---|---|---|---|---|---|---|---|---|
| | n | MRI measure OR | p= | Model $pR^2$ | MRI measure $pR^2$ | Age and gender $pR^2$ | n | OR | p= | Model $pR^2$ | MRI measure $pR^2$ | Age and gender $pR^2$ | n | OR | p= | Model $pR^2$ | MRI measure $pR^2$ | Age, gender $pR^2$ |
| 0 year | 103 | 1.04 (1.01-1.07) | 0.004* | 0.177 | 0.142 | 0.035 | 87 | 1.22 (0.77-1.93) | 0.409 | 0.032 | 0.012 | 0.02 | 60 | 1.05 (0.56-1.95) | 0.880 | 0.031 | 0 | 0.031 |
| 1 year | 86 | 1.07 (1.03-1.11) | 0.001* | 0.354 | 0.274 | 0.08 | 76 | 1.55 (0.90-2.65) | 0.112 | 0.107 | 0.075 | 0.032 | 52 | 1.13 (0.62-2.02) | 0.696 | 0.043 | 0.002 | 0.041 |
| 5 year | 81 | 1.06 (1.03-1.09) | <0.001* | 0.485 | 0.377 | 0.108 | 70 | 1.58 (0.96-2.59) | 0.072 | 0.126 | 0.124 | 0.002 | 70 | 3.31 (1.56-7.04) | 0.002* | 0.301 | 0.221 | 0.08 |
| 10 year | 62 | 1.04 (1.02-1.07) | 0.001* | 0.489 | 0.333 | 0.156 | 61 | 1.93 (1.24-3.00) | 0.004* | 0.321 | 0.277 | 0.044 | 53 | 1.39 (0.65-2.97) | 0.398 | 0.108 | 0.048 | 0.06 |
| 14 year | 52 | 1.03 (1.01-1.05) | 0.001* | 0.482 | 0.366 | 0.116 | 47 | 2.49 (1.42-4.37) | 0.001* | 0.508 | 0.498 | 0.01 | 47 | 2.60 (1.13-6.02) | 0.025* | 0.224 | 0.149 | 0.075 |
| 20 year | 71 | 1.02 (1.01-1.04) | 0.001* | 0.305 | 0.271 | 0.034 | 70 | 1.62 (1.21-2.17) | 0.001* | 0.263 | 0.262 | 0.001 | 64 | 7.19 (1.87-27.78) | 0.004* | 0.260 | 0.26 | 0 |

**Table 4b – Longitudinal models adjusted for baseline predicting 30-year SPMS outcomes**
*Adjusted for baseline total lesion counts, age and gender*

| Timepoint | Total Lesion Counts | | | | | | TVW | | | | | | MEDW | | | | | |
|---|---|---|---|---|---|---|---|---|---|---|---|---|---|---|---|---|---|---|
| | n | OR | p= | Model $pR^2$ | MRI measure $pR^2$ | Age and gender $pR^2$ | n | OR | p= | Model $pR^2$ | MRI measure $pR^2$ | Age and gender $pR^2$ | n | OR | p= | Model $pR^2$ | MRI measure $pR^2$ | Age, gender $pR^2$ |
| 1 year | 86 | 1.10 (1.01-1.21) | 0.038* | 0.360 | 0.278 | 0.082 | 73 | 1.37 (0.77-2.42) | 0.283 | 0.105 | 0.071 | 0.034 | 41 | 1.72 (0.66-4.55) | 0.266 | 0.148 | 0.113 | 0.035 |
| 5 year | 81 | 1.09 (1.04-1.14) | <0.001* | 0.516 | 0.405 | 0.111 | 67 | 1.58 (0.90-2.77) | 0.113 | 0.118 | 0.089 | 0.029 | 50 | 5.10 (1.78-14.49) | 0.002* | 0.391 | 0.326 | 0.065 |
| 10 year | 62 | 1.04 (1.01-1.08) | 0.011* | 0.489 | 0.336 | 0.153 | 54 | 1.98 (1.20-3.25) | 0.007* | 0.321 | 0.279 | 0.042 | 32 | 1.87 (0.55-6.37) | 0.315 | 0.060 | 0.054 | 0.006 |
| 14 year | 52 | 1.03 (1.01-1.05) | 0.008* | 0.486 | 0.372 | 0.114 | 46 | 2.55 (1.43-4.55) | 0.001* | 0.508 | 0.497 | 0.011 | 47 | 2.77 (0.82-9.35) | 0.101 | 0.190 | 0.153 | 0.037 |
| 20 year | 71 | 1.02 (1.01-1.04) | 0.001* | 0.307 | 0.274 | 0.033 | 61 | 1.65 (1.20-2.27) | 0.002* | 0.300 | 0.296 | 0.004 | 64 | 8.85 (1.44-55.56) | 0.018* | 0.290 | 0.29 | 0 |

## Table 5 – Lesion count models (separated by location) predicting 30-year SPMS outcomes

**Table 5a – Cross-sectional models predicting 30-year SPMS outcomes**
*Adjusted for age and gender*

| Timepoint | PV lesions | | | | | | DWM lesions | | | | | |
|---|---|---|---|---|---|---|---|---|---|---|---|---|
| | n | MRI measure OR | p= | Model $pR^2$ | MRI measure $pR^2$ | Age and gender $pR^2$ | n | OR | p= | Model $pR^2$ | MRI measure $pR^2$ | Age and gender $pR^2$ |
| 0 year | 97 | 1.17 (1.04-1.31) | 0.007* | 0.146 | 0.122 | 0.024 | 97 | 1.06 (1.02-1.10) | 0.006* | 0.170 | 0.133 | 0.037 |
| 1 year | 82 | 1.25 (1.10-1.42) | <0.001* | 0.310 | 0.252 | 0.058 | 82 | 1.10 (1.04-1.17) | 0.001* | 0.328 | 0.266 | 0.062 |
| 5 year | 81 | 1.34 (1.16-1.54) | <0.001* | 0.502 | 0.374 | 0.128 | 81 | 1.07 (1.03-1.11) | <0.001* | 0.441 | 0.342 | 0.099 |
| 10 year | 62 | 1.29 (1.12-1.48) | <0.001* | 0.545 | 0.356 | 0.189 | 62 | 1.06 (1.02-1.10) | 0.001* | 0.450 | 0.317 | 0.133 |
| 14 year | 52 | 1.22 (1.09-1.37) | <0.001* | 0.553 | 0.403 | 0.150 | 52 | 1.05 (1.02-1.08) | 0.001* | 0.429 | 0.324 | 0.105 |
| 20 year | 71 | 1.14 (1.07-1.22) | <0.001* | 0.363 | 0.318 | 0.045 | 71 | 1.03 (1.01-1.05) | 0.002* | 0.248 | 0.22 | 0.028 |

| Timepoint | JC lesions | | | | | | IT lesions | | | | | |
|---|---|---|---|---|---|---|---|---|---|---|---|---|
| | n | MRI measure OR | p= | Model $pR^2$ | MRI measure $pR^2$ | Age and gender $pR^2$ | n | OR | p= | Model $pR^2$ | MRI measure $pR^2$ | Age and gender $pR^2$ |
| 0 year | 97 | 1.54 (1.13-2.11) | 0.007* | 0.157 | 0.119 | 0.038 | 96 | 3.03 (1.48-6.20) | 0.002* | 0.259 | 0.237 | 0.022 |
| 1 year | 82 | 1.49 (1.11-2.01) | 0.008* | 0.222 | 0.141 | 0.081 | 82 | 5.10 (2.01-12.93) | <0.001* | 0.445 | 0.417 | 0.028 |
| 5 year | 81 | 1.24 (1.06-1.46) | 0.007* | 0.261 | 0.152 | 0.109 | 78 | 2.30 (1.39-3.81) | 0.001* | 0.417 | 0.358 | 0.059 |
| 10 year | 61 | 1.09 (1.01-1.17) | 0.025* | 0.218 | 0.076 | 0.142 | 55 | 1.96 (1.18-3.26) | 0.01* | 0.474 | 0.298 | 0.176 |
| 14 year | 50 | 1.10 (1.02-1.18) | 0.016* | 0.269 | 0.163 | 0.106 | 47 | 1.90 (1.25-2.89) | 0.003* | 0.505 | 0.354 | 0.151 |
| 20 year | 69 | 1.08 (1.01-1.15) | 0.021* | 0.147 | 0.118 | 0.029 | 64 | 1.45 (1.12-1.89) | 0.005* | 0.299 | 0.258 | 0.041 |

**Table 5b – Longitudinal models adjusted for baseline predicting 30-year SPMS outcomes**
*Adjusted for baseline lesion counts (with respect to location), age and gender*

| Timepoint | PV lesions | | | | | | DWM lesions | | | | | |
|---|---|---|---|---|---|---|---|---|---|---|---|---|
| | n | MRI measure OR | p= | Model $pR^2$ | MRI measure $pR^2$ | Age and gender $pR^2$ | n | OR | p= | Model $pR^2$ | MRI measure $pR^2$ | Age and gender $pR^2$ |
| 1 year | 81 | 1.53 (1.12-2.09) | 0.008* | 0.335 | 0.335 | 0.000 | 81 | 1.16 (1.01-1.32) | 0.035* | 0.475 | 0.281 | 0.055 |
| 5 year | 80 | 1.60 (1.25-2.03) | <0.001* | 0.565 | 0.449 | 0.116 | 80 | 1.12 (1.04-1.20) | 0.002* | 0.336 | 0.389 | 0.086 |
| 10 year | 61 | 1.31 (1.11-1.55) | 0.002* | 0.556 | 0.39 | 0.166 | 61 | 1.06 (1.01-1.12) | 0.016* | 0.461 | 0.353 | 0.108 |
| 14 year | 51 | 1.25 (1.09-1.43) | 0.001* | 0.571 | 0.455 | 0.116 | 51 | 1.04 (1.01-1.08) | 0.025* | 0.464 | 0.384 | 0.080 |
| 20 year | 67 | 1.17 (1.07-1.28) | <0.001* | 0.402 | 0.362 | 0.040 | 67 | 1.03 (1.01-1.05) | 0.006* | 0.276 | 0.243 | 0.033 |

| Timepoint | JC lesions | | | | | | IT lesions | | | | | |
|---|---|---|---|---|---|---|---|---|---|---|---|---|
| | n | MRI measure OR | p= | Model $pR^2$ | MRI measure $pR^2$ | Age and gender $pR^2$ | n | OR | p= | Model $pR^2$ | MRI measure $pR^2$ | Age and gender $pR^2$ |
| 1 year | 81 | 1.44 (0.85-2.41) | 0.173 | 0.230 | 0.156 | 0.074 | 80 | 2.32 (0.79-6.81) | 0.126 | 0.474 | 0.45 | 0.024 |
| 5 year | 80 | 1.17 (1.00-1.38) | 0.056 | 0.292 | 0.195 | 0.097 | 77 | 2.10 (1.15-3.85) | 0.016* | 0.431 | 0.38 | 0.051 |
| 10 year | 60 | 1.04 (0.97-1.13) | 0.274 | 0.255 | 0.134 | 0.121 | 54 | 1.34 (0.92-1.97) | 0.132 | 0.570 | 0.447 | 0.123 |
| 14 year | 50 | 1.06 (0.98-1.14) | 0.129 | 0.339 | 0.234 | 0.105 | 47 | 1.66 (1.02-2.70) | 0.041* | 0.522 | 0.387 | 0.135 |
| 20 year | 66 | 1.07 (1.00-1.15) | 0.044* | 0.199 | 0.145 | 0.054 | 60 | 1.31 (0.98-1.75) | 0.070 | 0.405 | 0.368 | 0.037 |

## Table 6 – Clinical measures models predicting 30-year SPMS outcomes

**Table 6a – Relapse activity models predicting 30-year SPMS outcomes**
***Adjusted for age and gender***

| Timepoint | n | Relapse activity OR | p= | Model $pR^2$ | Relapse activity $pR^2$ | Age and gender $pR^2$ |
|---|---|---|---|---|---|---|
| **0-5 years** | **95** | **1.81 (1.29-2.54)** | **<0.001*** | **0.251** | **0.213** | **0.038** |
| **5-10 years** | **87** | **2.57 (1.56-4.22)** | **<0.001*** | **0.260** | **0.246** | **0.014** |
| **10-20 years** | **94** | **1.47 (1.04-2.08)** | **0.029*** | **0.096** | **0.058** | **0.038** |

**Table 6b – EDSS models predicting 30-year SPMS outcomes**
***Adjusted for age and gender***

| Timepoint | n | EDSS OR | p= | Model $pR^2$ | EDSS $pR^2$ | Age and gender $pR^2$ |
|---|---|---|---|---|---|---|
| 0 year | 100 | 1.23 (0.89-1.69) | 0.207 | 0.051 | 0.023 | 0.028 |
| 1 year (nadir) | 105 | 1.25 (0.92-1.71) | 0.154 | 0.058 | 0.045 | 0.013 |
| **5 year** | **105** | **1.89 (1.38-2.59)** | **<0.001*** | **0.307** | **0.303** | **0.004** |
| **10 year** | **106** | **2.46 (1.71-3.53)** | **<0.001*** | **0.512** | **0.501** | **0.011** |
| **14 year** | **106** | **2.13 (1.58-2.87)** | **<0.001*** | **0.519** | **0.503** | **0.016** |
| **20 year** | **107** | **2.83 (1.93-4.17)** | **<0.001*** | **0.694** | **0.688** | **0.006** |

## Table 7 – Additional linear atrophy models predicting 30-year SPMS outcomes

**Table 7a – Models considering absolute difference in linear atrophy measures from baseline predicting 30-year SPMS outcomes**
***Adjusted for age and gender***

| Timepoint | TVW | | | | | | MEDW | | | | | |
|---|---|---|---|---|---|---|---|---|---|---|---|---|
| | n | MRI measure OR | p= | Model $pR^2$ | MRI measure $pR^2$ | Age and gender $pR^2$ | n | OR | p= | Model $pR^2$ | MRI measure $pR^2$ | Age and gender $pR^2$ |
| 5 year | 67 | 1.32 (0.83-2.09) | 0.241 | 0.081 | 0.046 | 0.035 | **50** | **0.29 (0.12-0.67)** | **0.004*** | **0.344** | **0.281** | **0.063** |
| 10 year | **54** | **1.67 (1.13-2.46)** | **0.01*** | **0.239** | **0.199** | **0.040** | 32 | 0.61 (0.20-1.83) | 0.374 | 0.051 | 0.046 | 0.005 |
| 14 year | **46** | **2.34 (1.39-3.96)** | **0.001*** | **0.468** | **0.456** | **0.012** | 28 | 0.45 (0.15-1.33) | 0.150 | 0.125 | 0.093 | 0.032 |
| 20 year | **61** | **1.51 (1.15-2.00)** | **0.003*** | **0.239** | **0.225** | **0.014** | 39 | 0.57 (0.24-1.37) | 0.209 | 0.074 | 0.066 | 0.008 |

**Table 7b – Models considering rates of linear atrophy (mm/year x100)* from baseline predicting 30-year SPMS outcomes**
***Adjusted for age and gender***

| Timepoint | TVW | | | | | | MEDW | | | | | |
|---|---|---|---|---|---|---|---|---|---|---|---|---|
| | n | MRI measure OR | p= | Model $pR^2$ | MRI measure $pR^2$ | Age and gender $pR^2$ | n | OR | p= | Model $pR^2$ | MRI measure $pR^2$ | Age and gender $pR^2$ |
| 5 year | 67 | 1.01 (0.99-1.04) | 0.241 | 0.081 | 0.046 | 0.035 | **50** | **0.94 (0.90-0.98)** | **0.004*** | **0.344** | **0.281** | **0.063** |
| 10 year | **54** | **1.05 (1.01-1.09)** | **0.01*** | **0.239** | **0.199** | **0.040** | 32 | 0.95 (0.85-1.06) | 0.374 | 0.051 | 0.046 | 0.005 |
| 14 year | **46** | **1.13 (1.05-1.21)** | **0.001*** | **0.468** | **0.456** | **0.012** | 28 | 0.89 (0.77-1.04) | 0.150 | 0.125 | 0.093 | 0.032 |
| 20 year | **61** | **1.09 (1.03-1.15)** | **0.003*** | **0.239** | **0.225** | **0.014** | 39 | 0.89 (0.75-1.07) | 0.209 | 0.074 | 0.066 | 0.008 |

$$Rate\ of\ atrophy = \frac{Linear\ atrophy\ measure\ at\ a\ given\ time\ time\ point - Linear\ atrophy\ measure\ at\ baseline}{Time\ (years\ from\ baseline\ measure)}\ X\ 100$$

## Table 8 – Total lesion count and linear atrophy models predicting 30-year MS-related mortality outcomes

**Table 8a – Cross-sectional models predicting 30-year MS-related mortality outcomes**
*Adjusted for age and gender*

| Timepoint | Total Lesion Counts | | | | | | TVW | | | | | | MEDW | | | | | |
|---|---|---|---|---|---|---|---|---|---|---|---|---|---|---|---|---|---|---|
| | n | MRI measure OR | p= | Model $pR^2$ | MRI measure $pR^2$ | Age and gender $pR^2$ | n | OR | p= | Model $pR^2$ | MRI measure $pR^2$ | Age and gender $pR^2$ | n | OR | p= | Model $pR^2$ | MRI measure $pR^2$ | Age, gender $pR^2$ |
| 0 year | **103** | **1.03 (1.01-1.06)** | **0.006*** | **0.197** | **0.099** | **0.098** | 87 | 1.00 (0.54-1.83) | 0.989 | 0.067 | 0 | 0.067 | 60 | 1.37 (0.55-3.40) | 0.496 | 0.256 | 0.001 | 0.255 |
| 1 year | **86** | **1.04 (1.01-1.07)** | **0.005*** | **0.243** | **0.131** | **0.112** | 76 | 1.10 (0.64-1.90) | 0.721 | 0.112 | 0.013 | 0.099 | **52** | **0.39 (0.15-0.99)** | **0.048*** | **0.302** | **0.103** | **0.199** |
| 5 year | **81** | **1.03 (1.01-1.05)** | **0.001*** | **0.379** | **0.228** | **0.151** | 70 | 1.08 (0.62-1.87) | 0.797 | 0.207 | 0.046 | 0.161 | 70 | 0.92 (0.38-2.22) | 0.845 | 0.198 | 0.002 | 0.196 |
| 10 year | **62** | **1.04 (1.01-1.06)** | **0.004*** | **0.560** | **0.162** | **0.398** | 61 | 1.30 (0.90-1.86) | 0.164 | 0.254 | 0.079 | 0.175 | 53 | 1.24 (0.36-4.29) | 0.737 | 0.342 | 0.019 | 0.323 |
| 14 year | **52** | **1.03 (1.01-1.05)** | **0.013*** | **0.480** | **0.144** | **0.336** | **47** | **1.70 (1.06-2.73)** | **0.028*** | **0.405** | **0.301** | **0.104** | 47 | 2.59 (0.74-9.00) | 0.137 | 0.325 | 0.055 | 0.270 |
| 20 year | **71** | **1.03 (1.01-1.05)** | **0.004*** | **0.477** | **0.161** | **0.316** | **70** | **1.99 (1.19-3.35)** | **0.009*** | **0.524** | **0.377** | **0.147** | **64** | **17.2 (2.28-125.00)** | **0.006*** | **0.509** | **0.416** | **0.093** |

**Table 8b – Longitudinal models adjusted for baseline predicting 30-year MS-related mortality outcomes**
*Adjusted for baseline, age and gender*

| Timepoint | Total Lesion Counts | | | | | | TVW | | | | | | MEDW | | | | | |
|---|---|---|---|---|---|---|---|---|---|---|---|---|---|---|---|---|---|---|
| | n | OR | p= | Model $pR^2$ | MRI measure $pR^2$ | Age and gender $pR^2$ | n | OR | p= | Model $pR^2$ | MRI measure $pR^2$ | Age and gender $pR^2$ | n | OR | p= | Model $pR^2$ | MRI measure $pR^2$ | Age, gender $pR^2$ |
| 1 year | 86 | 1.058 (0.99-1.13) | 0.088 | 0.249 | 0.134 | 0.115 | 73 | 1.08 (0.58-2.01) | 0.799 | 0.107 | 0.01 | 0.097 | 41 | 0.96 (0.26-3.52) | 0.951 | 0.292 | 0.054 | 0.238 |
| 5 year | **81** | **1.04 (1.01-1.07)** | **0.010*** | **0.400** | 0.254 | 0.146 | 67 | 1.18 (0.64-2.16) | 0.597 | 0.218 | 0.066 | 0.152 | 50 | 0.75 (0.24-2.36) | 0.619 | 0.372 | 0.022 | 0.350 |
| 10 year | **62** | **1.05 (1.01-1.09)** | **0.027*** | **0.566** | 0.168 | 0.398 | 54 | 1.29 (0.86-1.93) | 0.223 | 0.241 | 0.067 | 0.174 | 32 | 2.07 (0.20-21.3) | 0.538 | 0.692 | 0.05 | 0.642 |
| 14 year | 52 | 1.02 (0.99-1.06) | 0.237 | 0.488 | 0.146 | 0.342 | **46** | **1.68 (1.05-2.70)** | **0.030*** | **0.414** | **0.309** | **0.105** | 28 | 3.21 (0.28-37.0) | 0.351 | 0.629 | 0.052 | 0.577 |
| 20 year | **71** | **1.03 (1.01-1.06)** | **0.008*** | **0.481** | 0.168 | 0.313 | **61** | **1.98 (1.14-3.44)** | **0.016*** | **0.511** | **0.366** | **0.145** | - | - | - | - | - | - |

## Table 9 – Lesion count models (separated by location) predicting 30-year MS-related mortality outcomes

**Table 9a – Cross-sectional models predicting 30-year MS-related mortality outcomes**
***Adjusted for age and gender***

| Timepoint | PV lesions | | | | | | DWM lesions | | | | | |
|---|---|---|---|---|---|---|---|---|---|---|---|---|
| | n | MRI measure OR | p= | Model $pR^2$ | MRI measure $pR^2$ | Age and gender $pR^2$ | n | OR | p= | Model $pR^2$ | MRI measure $pR^2$ | Age and gender $pR^2$ |
| 0 year | **97** | **1.13 (1.01-1.26)** | **0.030*** | **0.162** | 0.087 | 0.075 | **97** | **1.04 (1.01-1.08)** | **0.015*** | **0.175** | 0.071 | 0.104 |
| 1 year | **82** | **1.17 (1.05-1.31)** | **0.006*** | **0.260** | 0.172 | 0.088 | **82** | **1.05 (1.01-1.09)** | **0.016*** | **0.211** | 0.096 | 0.115 |
| 5 year | **81** | **1.11 (1.02-1.20)** | **0.020*** | **0.237** | 0.124 | 0.113 | **81** | **1.04 (1.02-1.06)** | **<0.001*** | **0.365** | 0.215 | 0.150 |
| 10 year | **62** | **1.23 (1.07-1.40)** | **0.003*** | **0.485** | 0.103 | 0.382 | **62** | **1.04 (1.01-1.08)** | **0.006*** | **0.519** | 0.168 | 0.351 |
| 14 year | 52 | 1.09 (1.00-1.19) | 0.057 | 0.368 | 0.081 | 0.287 | **52** | **1.04 (1.01-1.07)** | **0.012*** | **0.462** | 0.150 | 0.312 |
| 20 year | **71** | **1.29 (1.08-1.53)** | **0.005*** | **0.637** | 0.258 | 0.379 | **71** | **1.04 (1.01-1.06)** | **0.008*** | **0.403** | 0.126 | 0.277 |

| Timepoint | JC lesions | | | | | | IT lesions | | | | | |
|---|---|---|---|---|---|---|---|---|---|---|---|---|
| | n | MRI measure OR | p= | Model $pR^2$ | MRI measure $pR^2$ | Age and gender $pR^2$ | n | OR | p= | Model $pR^2$ | MRI measure $pR^2$ | Age and gender $pR^2$ |
| 0 year | **97** | **1.37 (1.04-1.81)** | **0.027*** | **0.157** | **0.057** | **0.100** | **96** | **2.10 (1.29-3.43)** | **0.003*** | **0.298** | 0.201 | 0.097 |
| 1 year | 82 | 1.23 (0.98-1.54) | 0.074 | 0.159 | 0.035 | 0.124 | **78** | **2.26 (1.40-3.65)** | **<0.001*** | **0.450** | 0.345 | 0.105 |
| 5 year | 81 | 1.11 (0.98-1.25) | 0.096 | 0.227 | 0.085 | 0.142 | **82** | **1.83 (1.26-2.66)** | **0.001*** | **0.557** | 0.426 | 0.131 |
| 10 year | 61 | 1.07 (0.98-1.17) | 0.128 | 0.248 | 0.003 | 0.245 | **55** | **1.86 (1.23-2.82)** | **0.003*** | **0.695** | 0.363 | 0.332 |
| 14 year | 50 | 1.06 (0.97-1.16) | 0.214 | 0.289 | 0.009 | 0.280 | **47** | **2.75 (1.03-7.29)** | **0.043*** | **0.778** | 0.361 | 0.417 |
| 20 year | 69 | 1.06 (0.98-1.15) | 0.167 | 0.234 | 0.014 | 0.220 | **64** | **1.67 (1.15-2.43)** | **0.008*** | **0.572** | 0.240 | 0.332 |

**Table 9b – Longitudinal models adjusted for baseline predicting 30-year MS-related mortality outcomes**
***Adjusted for baseline lesion counts (with respect to location), age and gender***

| Timepoint | PV lesions | | | | | | DWM lesions | | | | | |
|---|---|---|---|---|---|---|---|---|---|---|---|---|
| | n | MRI measure OR | p= | Model $pR^2$ | MRI measure $pR^2$ | Age and gender $pR^2$ | n | OR | p= | Model $pR^2$ | MRI measure $pR^2$ | Age and gender $pR^2$ |
| 1 year | **81** | **1.45 (1.10-1.91)** | **0.008*** | **0.319** | 0.200 | 0.119 | 81 | 1.04 (0.94-1.15) | 0.422 | 0.209 | 0.096 | 0.113 |
| 5 year | **80** | **1.34 (1.10-1.63)** | **0.004*** | **0.354** | 0.185 | 0.169 | **80** | **1.05 (1.01-1.09)** | **0.007*** | **0.385** | 0.243 | 0.142 |
| 10 year | **61** | **1.20 (1.04-1.38)** | **0.015*** | **0.505** | 0.115 | 0.390 | **61** | **1.06 (1.01-1.11)** | **0.022*** | **0.535** | 0.187 | 0.348 |
| 14 year | 51 | 1.08 (0.95-1.23) | 0.242 | 0.376 | 0.092 | 0.284 | 51 | 1.03 (0.99-1.07) | 0.145 | 0.476 | 0.147 | 0.329 |
| 20 year | **67** | **1.28 (1.06-1.54)** | **0.011*** | **0.642** | 0.254 | 0.388 | **67** | **1.04 (1.01-1.07)** | **0.011*** | **0.405** | 0.137 | 0.268 |

| Timepoint | JC lesions | | | | | | IT lesions | | | | | |
|---|---|---|---|---|---|---|---|---|---|---|---|---|
| | n | MRI measure OR | p= | Model $pR^2$ | MRI measure $pR^2$ | Age and gender $pR^2$ | n | OR | p= | Model $pR^2$ | MRI measure $pR^2$ | Age and gender $pR^2$ |
| 1 year | 81 | 1.23 (0.72-2.10) | 0.444 | 0.158 | 0.038 | 0.12 | **80** | **1.97 (1.15-3.39)** | **0.014*** | **0.456** | **0.359** | **0.097** |
| 5 year | 80 | 1.14 (0.97-1.34) | 0.111 | 0.238 | 0.097 | 0.141 | **77** | **1.64 (1.14-2.37)** | **0.008*** | **0.569** | **0.428** | **0.141** |
| 10 year | 60 | 1.05 (0.94-1.17) | 0.391 | 0.263 | 0.005 | 0.258 | **54** | **1.68 (1.03-2.75)** | **0.039*** | **0.702** | **0.385** | **0.317** |
| 14 year | 50 | 1.03 (0.91-1.15) | 0.659 | 0.341 | 0.044 | 0.297 | 47 | 1.84 (0.79-4.30) | 0.158 | 0.794 | 0.398 | 0.396 |
| 20 year | 66 | 1.06 (0.96-1.16) | 0.249 | 0.235 | 0.017 | 0.218 | 60 | 1.51 (0.91-2.51) | 0.114 | 0.575 | 0.323 | 0.252 |

# Table 10 – Total lesion count and atrophy models predicting 30-year EDSS ≥3.5 outcomes

**Table 10a – Cross-sectional models predicting 30-year EDSS ≥3.5 outcomes**
***Adjusted for age and gender***

| Timepoint | Total Lesion Counts | | | | | | TVW | | | | | | MEDW | | | | | |
|---|---|---|---|---|---|---|---|---|---|---|---|---|---|---|---|---|---|---|
| | n | MRI measure OR | p= | Model $pR^2$ | MRI measure $pR^2$ | Age and gender $pR^2$ | n | OR | p= | Model $pR^2$ | MRI measure $pR^2$ | Age and gender $pR^2$ | n | OR | p= | Model $pR^2$ | MRI measure $pR^2$ | Age, gender $pR^2$ |
| 0 year | **103** | **1.04 (1.01-1.07)** | **0.005*** | **0.169** | **0.034** | **0.135** | 87 | 1.06 (0.67-1.66) | 0.807 | 0.021 | 0.001 | 0.020 | 60 | 0.79 (0.42-1.46) | 0.447 | 0.031 | 0.016 | 0.015 |
| 1 year | **86** | **1.07 (1.03-1.11)** | **0.001*** | **0.318** | **0.076** | **0.242** | 76 | 1.51 (0.89-2.57) | 0.129 | 0.095 | 0.066 | 0.029 | 52 | 0.96 (0.53-1.72) | 0.884 | 0.020 | 0.001 | 0.019 |
| 5 year | **81** | **1.06 (1.03-1.09)** | **<0.001*** | **0.489** | **0.111** | **0.378** | 70 | 1.37 (0.86-2.17) | 0.187 | 0.088 | 0.053 | 0.035 | **70** | **3.83 (1.73-8.47)** | **<0.001*** | **0.333** | **0.270** | **0.063** |
| 10 year | **62** | **1.05 (1.02-1.07)** | **<0.001*** | **0.543** | **0.197** | **0.346** | **61** | **2.11 (1.28-3.46)** | **0.003*** | **0.377** | **0.290** | **0.087** | 53 | 1.24 (0.59-2.62) | 0.574 | 0.151 | 0.049 | 0.102 |
| 14 year | **52** | **1.05 (1.02-1.07)** | **<0.001*** | **0.606** | **0.164** | **0.442** | **47** | **3.22 (1.58-6.58)** | **0.001*** | **0.592** | **0.536** | **0.056** | 47 | 2.00 (0.91-4.41) | 0.085 | 0.197 | 0.105 | 0.092 |
| 20 year | **71** | **1.03 (1.01-1.05)** | **<0.001*** | **0.389** | **0.025** | **0.364** | **70** | **2.14 (1.45-3.15)** | **<0.001*** | **0.426** | **0.423** | **0.003** | **64** | **13.5 (2.75-66.67)** | **0.001*** | **0.335** | **0.328** | **0.007** |

**Table 10b – Longitudinal models adjusted for baseline predicting 30-year EDSS ≥3.5 outcomes**
***Adjusted for baseline, age and gender***

| Timepoint | Total Lesion Counts | | | | | | TVW | | | | | | MEDW | | | | | |
|---|---|---|---|---|---|---|---|---|---|---|---|---|---|---|---|---|---|---|
| | n | OR | p= | Model $pR^2$ | MRI measure $pR^2$ | Age and gender $pR^2$ | n | OR | p= | Model $pR^2$ | MRI measure $pR^2$ | Age and gender $pR^2$ | n | OR | p= | Model $pR^2$ | MRI measure $pR^2$ | Age, gender $pR^2$ |
| 1 year | 81 | 1.06 (0.98-1.15) | 0.136 | 0.318 | 0.242 | 0.076 | 73 | 1.46 (0.80-2.67) | 0.213 | 0.084 | 0.055 | 0.029 | 41 | 1.28 (0.49-3.33) | 0.613 | 0.185 | 0.175 | 0.010 |
| 5 year | **86** | **1.10 (1.04-1.16)** | **<0.001*** | **0.526** | **0.413** | **0.113** | 67 | 1.42 (0.83-2.44) | 0.198 | 0.087 | 0.050 | 0.037 | **50** | **12.99 (2.79-62.50)** | **0.001*** | **0.565** | **0.526** | **0.039** |
| 10 year | **62** | **1.06 (1.02-1.10)** | **0.005*** | **0.553** | **0.366** | **0.187** | **54** | **2.23 (1.26-3.96)** | **0.006*** | **0.381** | **0.291** | **0.090** | 32 | 2.38 (0.59-9.52) | 0.220 | 0.199 | 0.189 | 0.010 |
| 14 year | **52** | **1.05 (1.02-1.08)** | **0.002*** | **0.607** | **0.442** | **0.165** | **46** | **3.52 (1.67-7.44)** | **<0.001*** | **0.618** | **0.549** | **0.069** | 28 | 2.92 (0.77-11.11) | 0.116 | 0.221 | 0.174 | 0.047 |
| 20 year | **71** | **1.03 (1.02-1.05)** | **<0.001*** | **0.397** | **0.375** | **0.022** | **61** | **2.25 (1.46-3.47)** | **<0.001*** | **0.476** | **0.463** | **0.013** | **39** | **21.73 (2.07-250.00)** | **0.010*** | **0.415** | **0.395** | **0.020** |

# Table 11 – Lesion count models (separated by location) predicting 30-year EDSS ≥3.5 outcomes

**Table 11a – Cross-sectional models predicting 30-year SPMS outcomes**
***Adjusted for age and gender***

| Timepoint | PV lesions | | | | | | DWM lesions | | | | | |
|---|---|---|---|---|---|---|---|---|---|---|---|---|
| | n | MRI measure OR | p= | Model $pR^2$ | MRI measure $pR^2$ | Age and gender $pR^2$ | n | OR | p= | Model $pR^2$ | MRI measure $pR^2$ | Age and gender $pR^2$ |
| 0 year | **97** | **1.16 (1.04-1.31)** | **0.01*** | **0.135** | **0.110** | **0.025** | **97** | **1.06 (1.02-1.11)** | **0.008*** | **0.166** | **0.130** | **0.036** |
| 1 year | **82** | **1.22 (1.08-1.38)** | **0.001*** | **0.263** | **0.209** | **0.054** | **82** | **1.10 (1.03-1.17)** | **0.003*** | **0.297** | **0.240** | **0.057** |
| 5 year | **81** | **1.28 (1.13-1.45)** | **<0.001*** | **0.432** | **0.314** | **0.118** | **81** | **1.08 (1.04-1.13)** | **<0.001*** | **0.470** | **0.366** | **0.104** |
| 10 year | **62** | **1.25 (1.11-1.40)** | **<0.001*** | **0.522** | **0.298** | **0.224** | **62** | **1.08 (1.03-1.12)** | **<0.001*** | **0.540** | **0.363** | **0.177** |
| 14 year | **52** | **1.22 (1.09-1.37)** | **<0.001*** | **0.576** | **0.378** | **0.198** | **52** | **1.08 (1.03-1.13)** | **<0.001*** | **0.619** | **0.455** | **0.164** |
| 20 year | **71** | **1.13 (1.06-1.20)** | **<0.001*** | **0.319** | **0.282** | **0.037** | **71** | **1.04 (1.02-1.07)** | **<0.001*** | **0.366** | **0.346** | **0.020** |

| Timepoint | JC lesions | | | | | | IT lesions | | | | | |
|---|---|---|---|---|---|---|---|---|---|---|---|---|
| | n | MRI measure OR | p= | Model $pR^2$ | MRI measure $pR^2$ | Age and gender $pR^2$ | n | OR | p= | Model $pR^2$ | MRI measure $pR^2$ | Age and gender $pR^2$ |
| 0 year | **97** | **1.50 (1.09-2.05)** | **0.012*** | **0.138** | **0.102** | **0.036** | **96** | **2.66 (1.34-5.29)** | **0.005*** | **0.221** | **0.196** | **0.025** |
| 1 year | **82** | **1.49 (1.09-2.05)** | **0.013*** | **0.206** | **0.132** | **0.074** | **82** | **4.09 (1.69-9.88)** | **0.002*** | **0.374** | **0.346** | **0.028** |
| 5 year | **81** | **1.25 (1.06-1.49)** | **0.009*** | **0.255** | **0.148** | **0.107** | **78** | **2.20 (1.33-3.67)** | **0.002*** | **0.381** | **0.316** | **0.065** |
| 10 year | **61** | **1.09 (1.01-1.17)** | **0.025*** | **0.264** | **0.072** | **0.192** | **55** | **2.02 (1.15-3.55)** | **0.014*** | **0.508** | **0.260** | **0.248** |
| 14 year | **50** | **1.10 (1.02-1.19)** | **0.018*** | **0.318** | **0.156** | **0.162** | **47** | **2.19 (1.33-3.61)** | **0.002*** | **0.592** | **0.350** | **0.242** |
| 20 year | **69** | **1.09 (1.01-1.16)** | **0.018*** | **0.162** | **0.137** | **0.025** | **64** | **1.67 (1.15-2.42)** | **0.007*** | **0.348** | **0.307** | **0.041** |

**Table 11b – Longitudinal models adjusted for baseline predicting 30-year SPMS outcomes**
***Adjusted for baseline lesion counts (with respect to location), age and gender***

| Timepoint | PV lesions | | | | | | DWM lesions | | | | | |
|---|---|---|---|---|---|---|---|---|---|---|---|---|
| | n | MRI measure OR | p= | Model $pR^2$ | MRI measure $pR^2$ | Age and gender $pR^2$ | n | OR | p= | Model $pR^2$ | MRI measure $pR^2$ | Age and gender $pR^2$ |
| 1 year | **81** | **1.34 (1.02-1.76)** | **0.036*** | **0.266** | **0.210** | **0.056** | 81 | 1.10 (0.97-1.25) | 0.128 | 0.296 | 0.244 | 0.052 |
| 5 year | **80** | **1.42 (1.17-1.73)** | **<0.001*** | **0.469** | **0.363** | **0.106** | **80** | **1.16 (1.06-1.26)** | **<0.001*** | **0.517** | **0.426** | **0.091** |
| 10 year | **61** | **1.25 (1.09-1.43)** | **0.002*** | **0.525** | **0.323** | **0.202** | **61** | **1.10 (1.04-1.18)** | **0.002*** | **0.564** | **0.436** | **0.128** |
| 14 year | **51** | **1.25 (1.09-1.43)** | **0.002*** | **0.584** | **0.418** | **0.166** | **51** | **1.09 (1.03-1.15)** | **0.002*** | **0.633** | **0.505** | **0.128** |
| 20 year | **67** | **1.15 (1.06-1.25)** | **<0.001*** | **0.342** | **0.310** | **0.032** | **67** | **1.05 (1.02-1.07)** | **<0.001*** | **0.401** | **0.382** | **0.019** |

| Timepoint | JC lesions | | | | | | IT lesions | | | | | |
|---|---|---|---|---|---|---|---|---|---|---|---|---|
| | n | MRI measure OR | p= | Model $pR^2$ | MRI measure $pR^2$ | Age and gender $pR^2$ | n | OR | p= | Model $pR^2$ | MRI measure $pR^2$ | Age and gender $pR^2$ |
| 1 year | 81 | 1.58 (0.87-2.89) | 0.136 | 0.215 | 0.146 | 0.069 | 80 | 1.96 (0.76-5.05) | 0.166 | 0.405 | 0.379 | 0.026 |
| 5 year | 80 | 1.19 (0.99-1.42) | 0.059 | 0.274 | 0.179 | 0.095 | **77** | **2.13 (1.15-3.91)** | **0.015*** | **0.387** | **0.332** | **0.055** |
| 10 year | 60 | 1.05 (0.97-1.14) | 0.257 | 0.287 | 0.120 | 0.167 | 54 | 1.39 (0.86-2.22) | 0.177 | 0.568 | 0.376 | 0.192 |
| 14 year | 50 | 1.06 (0.98-1.15) | 0.155 | 0.381 | 0.223 | 0.158 | **47** | **2.08 (1.16-3.75)** | **0.014*** | **0.593** | **0.358** | **0.235** |
| 20 year | **66** | **1.08 (1.01-1.16)** | **0.033*** | **0.199** | **0.156** | **0.043** | **60** | **1.52 (1.05-2.22)** | **0.029*** | **0.398** | **0.353** | **0.045** |
